# Recurrent Transient Ischemic Attacks in a Patient with POEMS Syndrome

**DOI:** 10.1155/2014/158471

**Published:** 2014-03-04

**Authors:** A. Akyol, B. Nazliel, H. Z. Batur Caglayan, Y. Oner, G. Turkoz Sucak

**Affiliations:** ^1^Department of Neurology, Faculty of Medicine, Gazi University, 06500 Ankara, Turkey; ^2^Department of Radiology, Faculty of Medicine, Gazi University, 06500 Ankara, Turkey; ^3^Department of Hematology, Faculty of Medicine, Gazi University, 06500 Ankara, Turkey

## Abstract

A 47-year-old female with a prior history of POEMS (polyneuropathy, organomegaly, endocrinopathy, monoclonal gammopathy, and skin changes) syndrome was admitted with transient ischemic attacks complicated by dysarthria and right-sided hemiparesis. A blood survey indicated thrombocytosis and hyperfibrinogenemia while imaging of intracranial vasculature showed occlusion of the bilateral middle cerebral arteries. POEMS syndrome, of which arterial thromboses have been mentioned as a manifestation, is rarely accompanied by transient ischemic attacks. The pathophysiologic mechanism is yet unclear and needs further investigation.

## 1. Introduction

POEMS (polyneuropathy, organomegaly, endocrinopathy, monoclonal gammopathy, and skin changes) syndrome is a paraneoplastic disorder due to an underlying clonal plasma cell neoplasm. Sclerotic bone lesions, elevated vascular endothelial growth factor, presence of Castleman disease, clonal plasma cells, and sensorimotor polyradiculoneuropathy are the major diagnostic criteria of the syndrome among which the latter two are *sine qua non. *At least one of the minor criteria, which include organomegaly, edema, endocrinopathy, skin changes, thrombocytosis/polycythemia, weight loss, hyperhidrosis, restrictive lung disease, diarrhea, and papilledema, is also required to establish the diagnosis [1]. Arterial thromboses have also been mentioned as a manifestation of POEMS syndrome. In contrast to the relatively frequent involvement of coronary and lower limb arteries, thromboses in cerebrovascular circulation have rarely been reported.

## 2. Case Report

**Figure 1:**
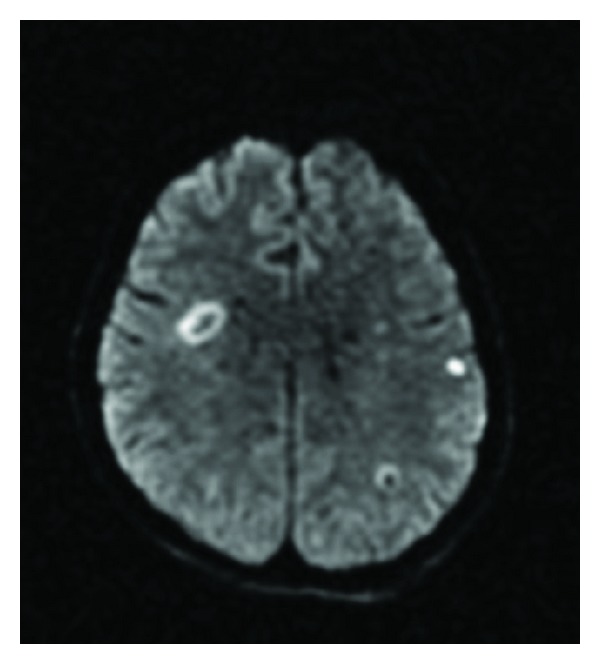
Diffusion-weighted image at the axial plane depicts bilateral ischemic foci at parietal lobes.

A 47-year-old female was admitted to our center with a 6-month history of transient ischemic attacks complicated with dysarthria and right-sided hemiparesis lasting 30 minutes. She experienced left-sided weakness on several occasions. She was describing as many as four to five attacks per day during the previous month. Her medical history was noteworthy for POEMS syndrome, which was diagnosed 2.5 months before her admission, hypertension, Hashimoto thyroiditis, and pulmonary embolism; and she was receiving calcium dobesilate, B complex vitamins, citalopram, trimetazidine, levothyroxine, warfarin, and acetyl salicylic acid daily and furosemide on alternate days. She had been diagnosed with diabetes mellitus and hyperlipidemia two years prior to admission to our center and had been treated with 30 mg gliclazide and 20 mg atorvastatin daily. A few months later she had discontinued her oral antidiabetic medication by her own will and was not restarted again because of good glycemic control despite discontinuation, with fasting glucose levels between 102 and 115 mg/dL (70–100 mg/dL) and postprandial values between 112 and 121 mg/dL (<140 mg/dL). She had received atorvastatin regularly for two years and the antihyperlipidemic treatment had been ceased recently by her physicians due to the low levels of total cholesterol (106 mg/dL; range: 110–200 mg/dL) and low density lipoprotein (52 mg/dL; range: 60–130 mg/dL). The physical examination findings included cachexia, plethora on the face, livedo reticularis on the chest and extremities, sclerodactyly, hepatomegaly, splenomegaly, and pretibial edema. A neurological examination revealed bilateral papilledema, dysarthria, right-sided hemiparesis, loss of sensation, and vibration in the lower limbs. Deep tendon reflexes were diminished in the upper limbs and abolic in the lower limbs, and her gait was ataxic. An electrocardiogram showed sinus rhythm, and a chest X-ray was consistent with pulmonary congestion. A complete blood count and serum chemistry results were within normal limits except for thrombocytosis (687 × 10^9^/L; range: 150–400 × 10^9^/L). Erythrocyte sedimentation rate (44 mm/h; range: 0–20 mm/h) was high, and C-reactive protein (6.27 mg/L; range: 0–6 mg/L) was slightly elevated. The levels of thyroid stimulating hormone (TSH) and anti-thyroid antibodies were high as well (TSH: 6.3 uIU/mL; anti-T: 28.3 IU/mL; anti-TPO: 6.7 IU/mL; range: 0.35–4.9 uIU/mL, 0–4.1 IU/mL, 0–5.6 IU/mL, resp.). She had hyperfibrinogenemia (418 mg/dL; range: 80–350 mg/dL) and an adequate INR level of 2.08 (range: 0.8–1.25). Lupus anticoagulant was negative and the baseline protein C (%75, range: 70–130), protein S (%97, range: 65–140), and antithrombin III activity (%90, range: 80–120) before the initiation of coumadin therapy (which was initiated one month prior to admission due to pulmonary embolism) were normal. Screenings for mutations of Factor V Leiden and activated protein C resistance were all negative, as well as the anti-nuclear and anti-phospholipid antibodies. A lumbar puncture revealed a high cerebrospinal fluid protein level (140 mg/dL, range: 15–40 mg/dL), while glucose and other electrolytes were within the normal range. A nerve conduction study showed diffuse bilateral sensorimotor neuropathy in the upper and lower extremities. On diffusion-weighted imaging (DWI) scans, acute ischemic foci on the left frontal and parietal area and T2 hyperintensities consistent with chronic ischemic disease on the right periventricular white matter were observed on magnetic resonance imaging (MRI) (Figure 1).

**Figure 2:**
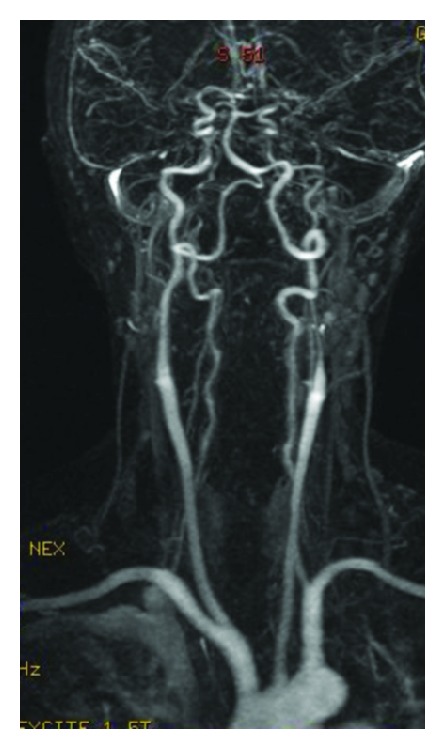
Supra-aortic MRA shows narrowing of the left internal carotid artery.

**Figure 3:**
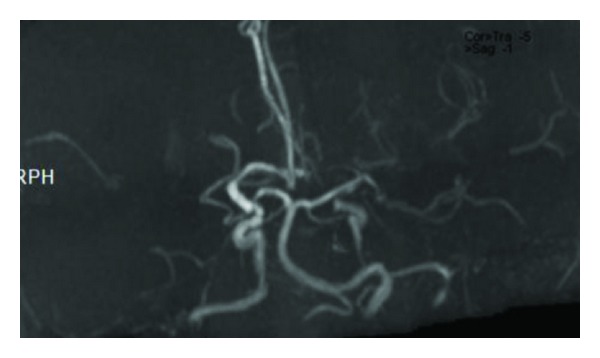
Cranial TOF MRA reveals bilateral occluded middle cerebral arteries.

Supra-aortic and intracranial MR angiography showed narrowing of the left internal carotid artery and occlusion of the bilateral middle cerebral arteries (Figures 2 and 3).

Transthoracic echocardiography revealed normal left ventricular volumes and ejection fraction, mild pericardial effusion, severe tricuspid regurgitation, and pulmonary hypertension (systolic pulmonary artery pressure was 80 mmHg). Neither intracardiac thrombus nor evidence of akinetic or hypokinetic segments, which may contribute to cardiac emboli, was detected on transthoracic echocardiography; and no finding which may indicate the presence of right to left shunt with Valsalva maneuver was present. She was closely monitored during hospitalization and observed to have more frequent attacks of right-sided weakness upon standing and walking, particularly on the days she took furosemide. The blood pressure in supine position (130/70 mmHg) and two minutes after standing (100/60 mmHg) was taken with the left arm supported at the elbow and the cuff at the heart level. She had decreases in her blood pressure of 30/10 mmHg and experienced the above mentioned ischemic attacks upon standing. These attacks were attributed to diuretic therapy and her diuretic regimen was halted. The presence of severe tricuspid insufficiency and increased pulmonary artery pressure (80 mmHg) which were considered as the activation of the disease process made us initiate Melphalan and Prednisolone (MP) for treatment. With cessation of diuretic therapy and following the commencement of steroids, she was observed to experience a lot of rare transient ischemic attacks and orthostatic hypotensive episodes. Digital subtraction angiography could not be performed as her condition deteriorated progressively. Her health status was not appropriate for extracranial (superficial temporal artery) intracranial (middle cerebral artery) bypass surgery and she was followed with medical treatment. After two cycles of MP treatment, she was rehospitalized again for autologous stem cell transplantation (ASCT). She was treated with cyclophosphamide as a conditioning regimen in order to collect stem cells. Unfortunately she succumbed to sudden cardiac death during the course of this hospitalization, right after collection of her stem cells.

## 3. Discussion

POEMS syndrome is a rare multisystem disorder, which might be associated with a constellation of clinical features including clubbing, weight loss, thrombocytosis, polycythemia, hyperhidrosis, hypertrichosis, pulmonary hypertension, restrictive lung disease, arthralgia, cardiomyopathy, fever, low vitamin B12 levels, and diarrhea. Arterial and venous thromboses have also been noted recently and appear to be a part of the syndrome. Most of the previous reports refer to lower limb and coronary artery involvement while ischemic cerebrovascular disease associated with POEMS syndrome has rarely been mentioned. The pathophysiological mechanisms causing the vascular events remain unclear. Our patient was a 47-year-old woman with frequent transient ischemic attacks who met all the criteria required for the diagnosis of POEMS syndrome. The etiological study for the ischemic attacks (complete blood count, blood chemistry, clotting times, screening for vasculitis, and thrombophilia) was unremarkable except thrombocytosis and hyperfibrinogenemia. Imaging of the supra-aortic and intracranial circulation showed occlusion at the origins of the bilateral middle cerebral arteries accompanied by an irregular contour. It is noteworthy to mention that all of the three cases with ischemic stroke of the patients with POEMS syndrome reported by Kang et al. had elevated fibrinogen levels. The authors have speculated that IL-6 induced hyperfibrinogenemia and inflammation could have a causal relationship with the vascular events [2]. However, not all patients with elevated cytokine levels have hyperfibrinogenemia and/or thrombosis.

This syndrome is rare and our knowledge depends on case reports or small retrospective cohorts. Namely, Lesprit et al. have reviewed the records of 20 patients with POEMS syndrome among which 4 had acute arterial obliteration including the one who developed a right internal carotid artery occlusion [3]. All of their four patients had elevated levels of interleukin- (IL-) 1*β*, tumor necrosis factor-*α*, and IL-6, and they claimed that nonimmunoglobulinemic mediators could be a possible cause for the arterial thrombosis in addition to thrombocytosis and microangiopathic vasculopathy. Lee et al. reported a 41-year-old male who presented with scleroderma-like skin changes who was diagnosed with POEMS syndrome after developing a right frontoparietal infarct due to narrowing of the right intracranial internal carotid artery and bilateral occlusion of the middle cerebral arteries [4]. The patient had occlusions of the superior mesenteric and left renal artery at the same time and died a short time later; therefore, the authors suggested that thrombosis is a bad prognostic factor associated with a catastrophic disease course. Garcia et al. investigated two patients with POEMS syndrome and recurrent ischemic attacks whose catheter cerebral angiogram findings indicated a nonatherosclerotic vasculopathic process despite the presence of atherosclerotic risk factors [5]. The largest retrospective cohort was reported from the Mayo Clinic by Dupont et al. Nineteen of their 208 patients with POEMS syndrome experienced ischemic stroke, and a subgroup analysis of 90 patients among whom nine had a stroke revealed no difference in the vascular endothelial growth factor or IL-6 levels or traditional stroke risk factors (such as age, diabetes, hypertension, smoking, and cardiac arrhythmia) between the stroke and nonstroke groups. However, they have not mentioned the fibrinogen levels in patients with and without stroke. The 5-year risk of ischemic stroke is 13.4% in their patients with POEMS syndrome, and the parameters associated with increased risk are thrombocytosis and evidence of plasma cell proliferation on bone marrow biopsy [6]. A subgroup of patients with increased fibrinogen levels might in fact be at risk of stroke. The inflammatory cytokines known to be elevated in POEMS syndrome or elevated fibrinogen levels might be the triggering factor for vascular inflammation and/or activation of coagulation or both in a subset of patients.

On the other hand, atherosclerosis due to diabetes mellitus, hypertension, and hyperlipidemia might as well have contributed to the bilateral middle cerebral artery occlusion in our patient though the ischemic attacks continued under appropriate antiaggregant and anticoagulant therapies.

In conclusion, we would like to emphasize the hemodynamic origin of orthostatic ischemic attacks particularly in patients using diuretics. Intracranial circulation should be investigated when extracranial circulation is insufficient to explain the cerebrovascular symptoms. POEMS syndrome should be considered in the differential diagnosis of the patients presenting with the combination of peripheral neuropathy and cerebrovascular events, particularly when a monoclonal protein in the serum is present.
